# Toluene Can Disrupt Rat Ovarian Follicullogenesis and Steroidogenesis and Induce Both Autophagy and Apoptosis

**DOI:** 10.3390/biology10111153

**Published:** 2021-11-09

**Authors:** Abdulkarem Alrezaki, Nouf Aldawood, Lamjed Mansour, Mukhtar Ahmed, Alexander V. Sirotkin, Saleh Alwasel, Abdel Halim Harrath

**Affiliations:** 1 Department of Zoology, College of Science, King Saud University, Riyadh 11451, Saudi Arabia; aayr1978@gmail.com (A.A.); n.daw333@gmail.com (N.A.); lmansour@ksu.edu.sa (L.M.); mahmed1@ksu.edu.sa (M.A.); salwasel@ksu.edu.sa (S.A.); 2 Department of Zoology and Anthropology, Constantine the Philosopher University in Nitra, 949 74 Nitra, Slovakia; asirotkin@ukf.sk

**Keywords:** toluene, reproductive toxicity, ovary, apoptosis, autophagy

## Abstract

**Simple Summary:**

Toluene, as one of the volatile organic solvents, has important industrial applications and can be used in a wide range of consumer and commercial products. It can be inhaled and absorbed easily by the human body and can therefore affect the individual’s health through damaging different body tissues, including the ovary. This organic solvent has been one of the most well-studied neurotoxins in recent decades. Studies reporting its effects on ovarian function are still limited. To advance our knowledge on the effect of toluene on female reproduction, an in vivo study using female Wistar rats was investigated. We found that toluene exposure affected ovarian structure and hormone balance by increasing progesterone and testosterone levels. In addition, it has disrupted most of the ovarian markers involved in granulosa cell proliferation and differentiation. Interestingly, Toluene exposure induced both apoptosis and autophagy, confirming the crosstalk between both mechanisms. The promising results of this study may contribute to the prevention of reproductive problems in society by raising the awareness about the use of this hydrocarbon.

**Abstract:**

Toluene has been shown to be highly toxic to humans and animals and can cause damage to various tissues. However, studies reporting its effects on ovarian function are still limited. In this study, we investigated the in vivo effect of toluene using female Wistar rats. We found that toluene exposure decreased ovarian weight and affected ovarian structure by increasing the number of abnormally growing follicles. Moreover, it significantly increased progesterone and testosterone levels. We also showed that toluene exposure decreased GDF-9 protein and its encoding gene. In addition, it inhibited the expression of most of the genes involved in granulosa cell proliferation and differentiation, such as *Insl3*, *ccnd2* and *actb*. The TUNEL assay showed that apoptosis occurred at the middle and high doses only (4000 and 8000 ppm, respectively), whereas no effect was observed at the low dose (2000 ppm). Interestingly, we showed that toluene exposure induced autophagy as LC3 protein and its encoding gene significantly increased for all doses of treatment. These results may suggest that the activation of autophagy at a low dose of exposure was to protect ovarian cells against death by inhibiting apoptosis, whereas its activation at high doses of exposure triggered apoptosis leading to cell death.

## 1. Introduction

Volatile organic solvents have important industrial applications and can be used in a wide range of consumer and commercial products, such as paints, cleaners and adhesives. Toluene, as one of these volatile organic compounds, represents one of the major precursors of secondary organic aerosols [1,2]. It can enter the environment mostly through emissions from automobiles and airplanes and losses during the marketing of gasoline, spills and cigarette smoke [3]. It has been identified as potentially dangerous to human health and the environment because it can be inhaled and absorbed by the human body [4]. Exposure to toluene can vary among countries and cities but the safe exposure levels are normally between 10 and 100 ppm, while the immediately harmful to life and health limit has been set at 500 ppm [5]. Nevertheless, the exposure may be at a high concentration abused by humans at a time point reaching, in some cases, 10,000 ppm [6,7].

Toluene exposure can affect an individual’s health [8] and can cause a variety of physiological problems. It can damage different body tissues, including the kidneys [9], liver tissue [8,10,11] and central nervous system [12]. This organic solvent has been one of the most well-studied neurotoxins in recent decades [13,14,15,16,17,18,19]. Indeed, toluene vapor exposure appears to have significant action on the dopaminergic system since it has increased the release of the neurotransmitter dopamine in both the whole brain and the striatum, while no effect was found on the secretion of norepinephrine and serotonin levels in the brain. The Norepinephrine, however, was found to increase in the medulla and midbrain whereas serotonin was increased in the cerebellum, medulla, and striatum [20]. In addition, toluene was described to have an antidepressant-like effect through an interaction with NMDA, GABAA and dopamine receptors [21]. Despite the advances, research into the molecular and cellular targets of inhaled toluene is still limited, owing to the widespread usage of this solvent as an inhalant. It has been defined that the inhaled toluene affected both voltage-gated and ligand-gated ion channels [22]. It can also trigger oxidative stress [23], apoptotic effects [24] and epigenetic changes that might have a long-term impact on gene expression and behavior [25]. 

Interestingly, toluene has been reported to cause renal and liver damage and toxicity in humans [26,27,28]. Animal study findings revealed that as toluene concentrations increased, fecundity in Drosophila melanogaster females and offspring survivability decreased, suggesting that toluene’s effects are dose-dependent and detrimental to fly development [29]. Different species of mammals, including rats, rabbits and cows, have been used in a variety of experiments to investigate the reproductive and developmental toxicity of toluene [30]. These studies conclusively showed that toluene exposure may adversely affect female reproduction. It can freely pass through the placenta to the fetus, thereby inducing fetotoxicity revealed by fetal morphological anomalies as well as a decrease in fecundity [31,32]. Moreover, inhalation of toluene has been shown to increase the incidence of maternal and fetal morbidity and embryonal malformations in women [33,34], cows [35] and rats [36,37]. However, in vivo evidence in support of the main mechanisms of the toluene effect on ovary function is still limited, particularly the induction of autophagic and apoptosis. In our recent in vitro studies, we demonstrated, using cultured healthy mare ovarian granulosa cells, that toluene inhibited viability, proliferation, apoptosis, progesterone, prostaglandin F and IGF-I release [38,39,40]. To advance our knowledge on the effect of toluene on female reproduction, an in vivo study using female Wistar rats is investigated. To our knowledge, this is the first study to test the in vivo mechanisms of the toluene effect on ovarian function, including the involvement of apoptosis and autophagy processes in ovarian cell toxicity.

## 2. Materials and Methods

### 2.1. Animal Treatment and Sampling 

This study was approved by the Scientific Research Ethics Committee at King Saud University (Reference No: KSU-SE-20-76). The Animal Welfare Center provided us with forty female Wistar rats weighing 200–250 g. They were held in single cages (22 to 24 °C) with a 12 h light/dark cycle and free access to food and water. The females were randomly divided into four groups of ten rats that were exposed to different concentrations of toluene for 28 days by inhalation for 30 min per day. The first group is composed of females that did not receive any toluene treatment. Rats in Group 2 were exposed to 2000 ppm toluene; rats in Group 3 were exposed to 4000 ppm toluene and rats in Group 4 were exposed to 8000 ppm toluene. After 28 days of treatment, animals were transferred individually to a transparent plastic box connected to a carbon dioxide tube at a flow rate of 10 L/hour for ten minutes. Blood samples were drawn from the heart and transferred directly to tubes containing an anticoagulant (EDTA) to obtain the serum after centrifugation. The ovaries were removed carefully, cleaned of adherent tissue and weighed. The sampled ovaries were labeled according to their origin (groups), and a portion of them were fixed in neutral buffered formalin (NBF) for classic histological study, immunostaining and TUNEL assay. A second portion was collected and stabilized in RNA later and stored at −80 °C for the molecular assays. 

### 2.2. Histology 

Ovarian samples were collected, weighed, and fixed in 10% neutral buffered formalin (NBF) for 24 h after 28 days of treatment. For classic histological analysis, samples were embedded in paraffin blocks that were serially cut into 5 µm-thick parts using a rotary microtome and stained with hematoxylin-eosin. For immunostaining and TUNEL assay, some blocks were cut into 3 µm-thick pieces. The follicular count for each ovary was calculated using the method mentioned in our previous research [41].

### 2.3. Hormones Analysis 

To obtain plasma, blood samples were collected into lithium heparin tubes and centrifuged at 3000 rpm for 10 min. The levels of progesterone, estradiol, testosterone and IGF-1 were measured using competitive enzyme-linked immunosorbent assays (ELISAs) as directed by the manufacturer (Vector Laboratories, New Jersey, USA).

### 2.4. TUNEL Assay 

The ovaries were fixed in 10% NBF for 24 h at room temperature for the TUNEL assay. Paraffin wax blocks were made, and 3 µm-thick paraffin parts were prepared and placed on coated slides. Dewaxed sections were rehydrated using normal methods and washed in phosphate-buffered saline (PBS). The tissue parts were then permeabilized in 0.1% Triton X-100 with 0.1% sodium citrate and incubated with a Proteinase K working solution at 37 °C for 15 min. TUNEL staining was performed according to the manufacturer’s instructions for the TMR red 12156792910 In Situ Cell Death Detection Kit (Roche Diagnostics, Mannheim, Germany). A positive control was created for one of the ovarian tissue sections, and a negative control was created by excluding the TdT from one of the other ovarian tissue sections. The nuclei were stained with Hoechst, and the labeled parts were examined with a Nikon TE 2000 fluorescence microscope using confocal microscopy (Nikon Co., Japan) to look for DNA fragmentation, chromatin condensation, and other apoptotic changes. The signal for TMR-red was analyzed by Zen 3.1 service (ZEN lite) and quantified using GraphPad Prism 9 program (GraphPad Software, San Diego, USA).

### 2.5. Immunostaining 

Slides containing tissue sections were placed on hotplates (60 °C) and dewaxed with xylene (2× 10 min). Then, they were rehydrated with decreasing concentrations of ethanol and washed twice with distilled water and with 1× PBS. The slides were removed from the washing process and dried by placing them diagonally on a surface containing dry tissues to ensure that the washing solution was removed. After drying, the slices were placed in a suitable container on the floor covered with layers of tissues moistened with water to reduce the rate of evaporation of the solution that the tissue sections would be treated with in the following stages. The tissue sections were then placed in 0.1% Triton X-100 with 0.1% sodium citrate. Blocking buffer (1% BSA in PBS) was used to block nonspecific staining (45 min at RT). The slides were placed into a humid box, and were then incubated with anti-GDF-9 (1:100 dilutions) and anti-LC-3 (1:100 dilutions) primary antibody. The humid box was closed carefully with tin paper and kept overnight at 4 °C on a flat balanced surface in the dark. On the second day, the slides were washed with 1× PBS, and were incubated with antirabbit FITC-conjugated secondary antibody (1:2000 dilutions (ab6717, Abcam, UK) for 45 min at RT in the dark. During the next step, slides were washed with PBS and then TE buffer before adding Hoechst solution (dilute 1:15,000, Hoechst 33342, life technologies, Grand Island, NJ, USA). Finally, the slices were washed, dried, and covered with cover slides, ensuring that the edges were covered with nail polish. Photomicrographs of GDF-9 and LC-3 specific signals and nuclear stains were captured with spinning disk confocal microscope from Zeiss (Germany). The immunofluorescence signal for protein expression was analyzed by Zen 3.1 service (ZEN lite, blue Edition, Germany) and quantified using GraphPad Prism 9 program (GraphPad Software, San Diego, CA, USA).

### 2.6. Analysis of Gene Expression 

RNA was extracted from ovarian tissues that had been previously stabilized with RNAlater (Qiagen, Westburg, The Netherlands). We used an RNeasy Mini Kit (Qiagen, Westburg, The Netherlands) with on-column DNase treatment with an RNase-Free DNase Package for RNA extraction (Qiagen). SYBR green and an Applied Biosynthesis 7500 Quick RT–PCR system (Carlsbad, CA, USA) were used to perform real-time PCR (RT–PCR) with the gene-specific primers described in Table 1. RT–PCR and several primer sets were used to obtain cDNA from these samples using an iScriptTM cDNA synthesis package (Applied Biosystem, Carlsbad, CA, USA). 

### 2.7. Statistical Analysis 

The mean and standard error of the mean were used to express the data (SEM). The statistical significance of the differences in mean values between the treatment and control groups was determined using GraphPad Prism version 5. For statistical comparisons, one-way analysis of variance (ANOVA) was used, followed by Tukey’s multiple comparison test. The difference was deemed statistically important when the *p* value was less than 0.05.

## 3. Results 

### 3.1. Effect of Toluene on Body Weight 

**Figure 1:**
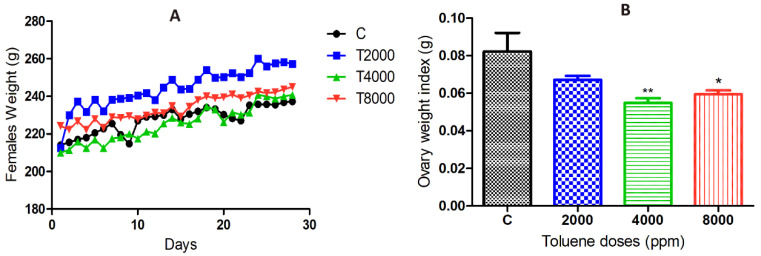
(**A**) Body weight changes over 28 days in the different groups exposed to toluene. (**B**) Ovarian weight changes in the toluene-exposed females compared to the control group. Values are means ± S.E.M. *, *p* < 0.05; **, *p* < 0.01.

The results showed a significant increase in body weight of the 2000 ppm-treated group compared to the control group, whereas no significant difference was found between the 4000 and 8000 ppm-treated groups compared to the control group (Figure 1A). 

### 3.2. Effect of Toluene on Ovary Weight 

Toluene exposure significantly reduced the ovarian weights of rats treated with doses of 4000 and 8000 ppm, whereas the dose of 2000 ppm did not affect ovarian weights (Figure 1B).

### 3.3. Effect of Toluene on the Number of Follicles 

**Figure 2:**
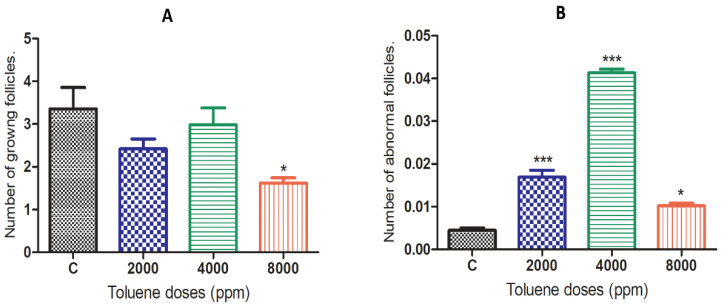
Effects of different doses of toluene on the (**A**) number of growing follicles and (**B**) abnormal follicles. Values are means ± S.E.M. *, *p* < 0.05; ***, *p* < 0.001.

The effects of toluene on the number of growing ovarian follicles and abnormal ovarian follicles are presented in Figure 2. The results showed that toluene significantly decreased the number of growing follicles only in the 8000 ppm-treated group compared to the control (Figure 2A). However, it significantly increased the number of abnormal follicles in ovaries from all treated groups, and the highest number was obtained in the group treated with 4000 ppm (Figure 2B). 

### 3.4. Effect of Toluene on Steroid Hormone Levels 

**Figure 3:**
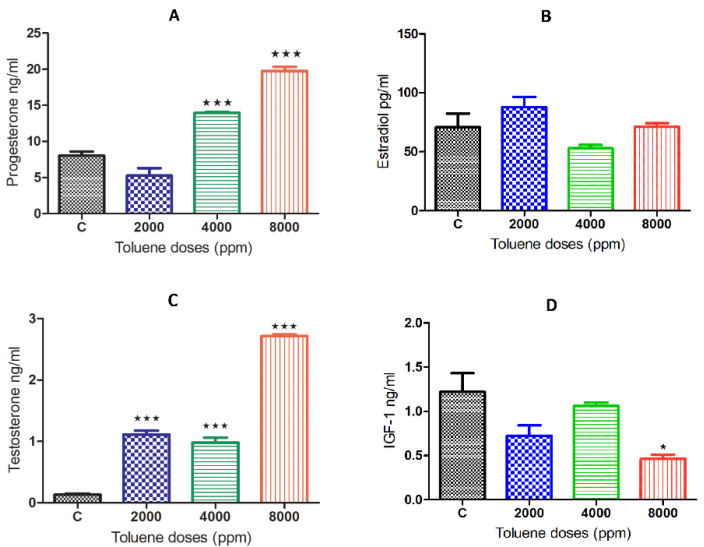
Serum hormone levels of female rats exposed to toluene compared to the control group. The levels of progesterone (**A**) significantly increased in females from the 4000 and 8000 groups, whereas testosterone levels (**C**) significantly increased among all treatments. However, no significant effect of toluene exposure was found on estradiol (**B**,**D**) IGF1. Values are means ± S.E.M. *, *p* < 0.05; ***, *p* < 0.001.

While there was no significant effect on estradiol (Figure 3B), exposure to toluene caused a significant increase in the secretion of the hormones progesterone and testosterone (Figure 3A,C) when compared to the control group. This increase was obtained for progesterone only at doses of 4000 and 8000 ppm, whereas it was observed among all treated groups for testosterone (Figure 3B,D). However, IGF-1 significantly decreased only with the high dose of treatment (Figure 3D).

### 3.5. Effect of Toluene on Gene Expression Levels

**Figure 4:**
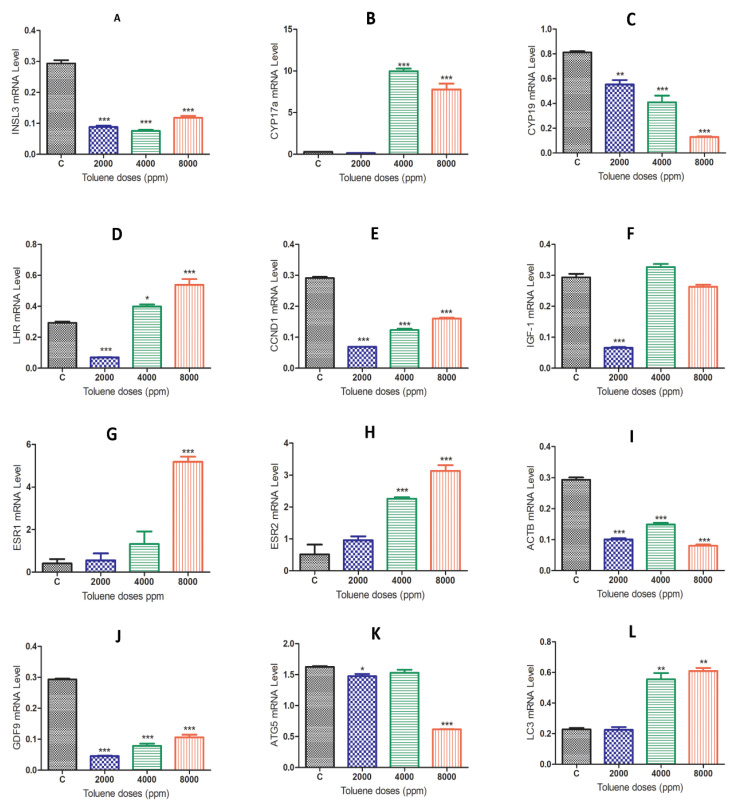
mRNA expression levels of different genes in the ovaries of rats in the treatment groups compared to the control group. (**A**), *INSL3*; (**B**), *CYP17a*; (**C**) *CYP19*; (**D**) *LHR*; (**E**) *CCND1*; (**F**) *IGF-1*; (**G**) *ESR1*; (**H**) *ESR2*; (**I**) *ACTB*; (**J**) *GDF-9*; (**K**) *ATG5*; (**L**) *LC-3*; Values are means ± S.E.M. *, *p* < 0.05; **, *p* < 0.01; ***, *p* < 0.001.

When comparing the gene expression results between the control group and the toluene-treated groups, we found that rats exposed to toluene at different doses significantly decreased the mRNA levels of the *Insl3*, *Cyp19*, *ccnd1*, *Igf-1*, *Actb*, *GDF-9* and *Atg5* genes (Figure 4A,C,E,F,I–K). Exposure to toluene significantly increased the expression of the *Cyp17a*, *Lhr*, *Esr2* and *Lc3* genes at 4000 and 8000 ppm (Figure 4B,D,H,L) and significantly increased the *Esr1* gene in the 2000 ppm-treated group (Figure 4G). 

### 3.6. Effect of Toluene on LC3 and GDF9 Protein Expression

#### 3.6.1. GDF9 Protein

**Figure 5:**
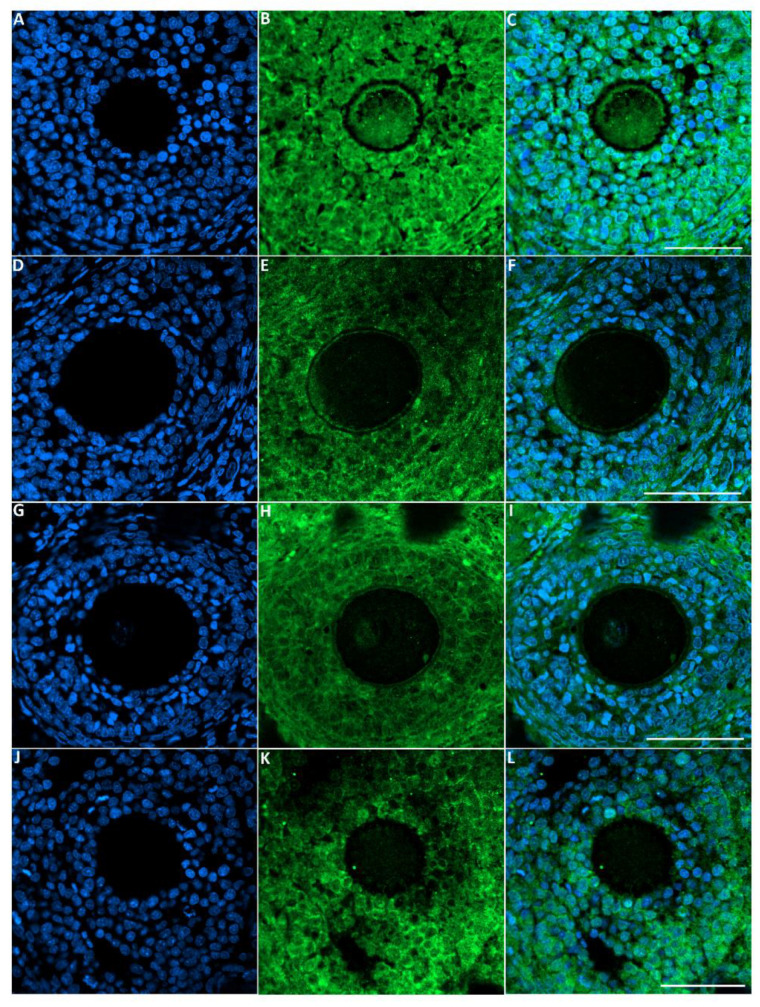
Representative images of GDF-9 immunofluorescence staining in rats of the different groups of the experiment. Control group (**A**–**C**), 2000 ppm-treated group (**D**–**F**), 4000 ppm-treated group (**G**–**I**) and 8000 ppm-treated group (**J**–**L**). Scale bars = 100 µm.

**Figure 6:**
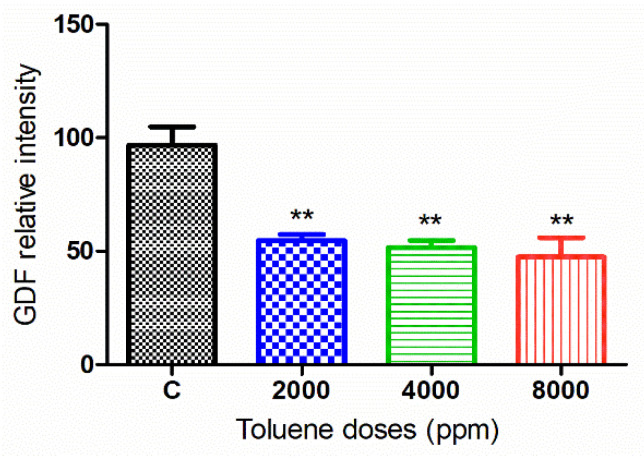
Relative fluorescence intensity of GDF-9 levels in the control and exposed groups. Values are means ± S.E.M. **, *p* < 0.01.

The green fluorescence intensity of GDF9 was significantly reduced in the ovaries of rats exposed to toluene at the different concentrations of treatment (2000, 4000 and 8000 ppm) compared to the control group (Figure 5 and Figure 6).

#### 3.6.2. LC3 Protein

**Figure 7:**
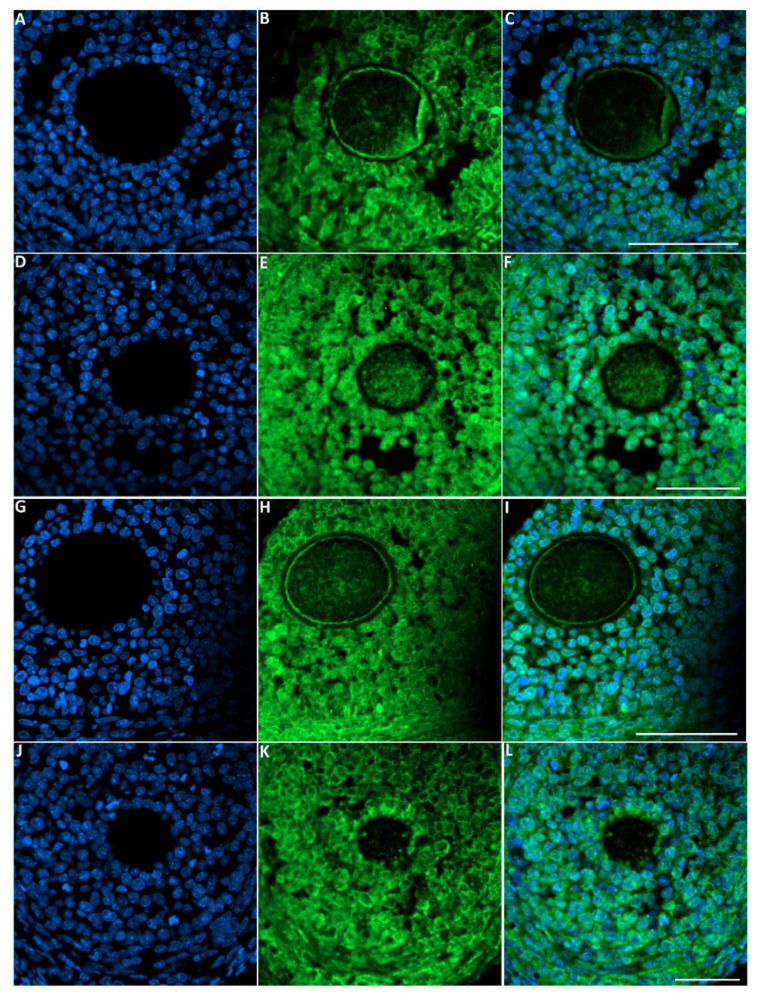
Representative images of LC-3 immunofluorescence staining in female rats that were exposed to toluene compared to the control. Control group (**A**–**C**), 2000 ppm-treated group (**D**–**F**), 4000 ppm-treated group (**G**–**I**) and 8000 ppm-treated group (**J**–**L**). Scale bars = 100 µm.

**Figure 8:**
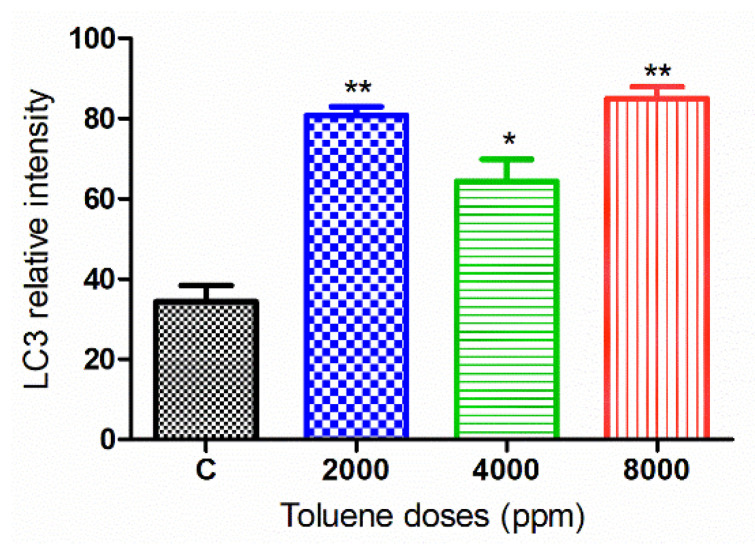
Relative fluorescence intensity of LC-3 levels in the control and exposed groups. Values are means ± S.E.M. *, *p* < 0.05; **, *p* < 0.01.

The green fluorescence intensity of LC3 was significantly increased in the ovaries of rats exposed to toluene at different concentrations (2000, 4000 and 8000 ppm) compared to the control group (Figure 7 and Figure 8).

### 3.7. DNA Damage in Cells Treated with Toluene

**Figure 9:**
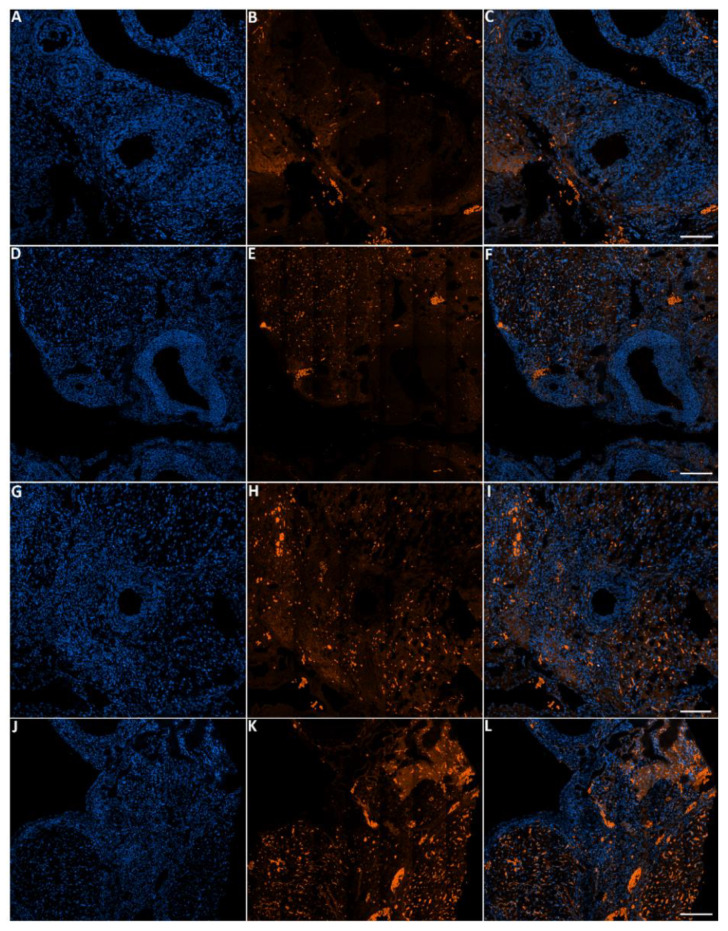
Representative images of apoptosis in ovaries from the control and treated groups stained with Hoechst and TMR red. Ovaries from control (**A**–**C**), ovaries from females that were exposed to 2000 ppm (**D**–**F**), 4000 ppm (**G**–**I**) and 8000 ppm (**J**–**L**). Scale bars = 100 µm.

**Figure 10:**
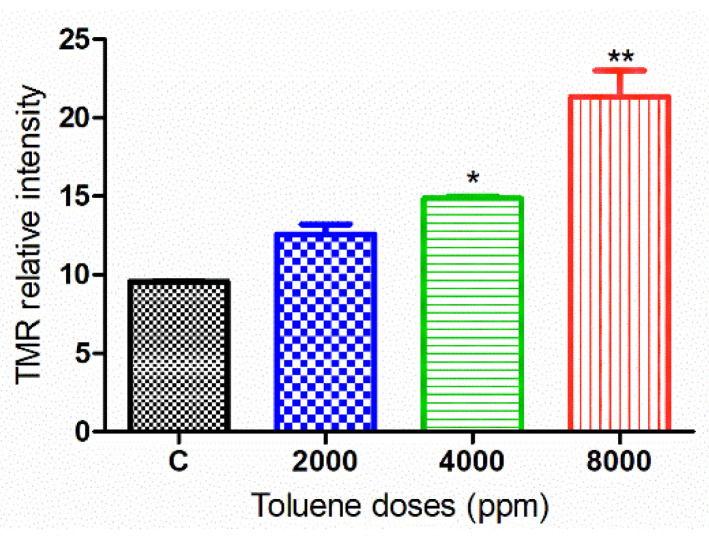
TMR-Red relative fluorescence intensity in the control and exposure groups. Values are means ± S.E.M. *, *p* < 0.05; **, *p* < 0.01.

Apoptosis was measured in ovarian tissues from various treatment groups (Figure 9). When comparing the treated groups to the control, the relative intensity of TMR-Red revealed a dose-dependent increase in apoptosis. However, this increase was not significant in ovaries from females treated with the dose 2000 ppm (Figure 10).

## 4. Discussion

Exposure to hazardous chemicals is currently the main occupational disease and the main clinical concern, especially when we know that the direct effect may affect the next generation [42,43]. Among these toxicants, toluene has been shown to cause multiple injuries and damage to different tissues of the body. These include cancer and many other chronic diseases, such as shortness of breath and leukemia [3]. However, studies reporting its effects on ovarian function are still limited. In our previous in vitro studies, we demonstrated that toluene inhibited ovarian cell viability, proliferation and estrogen release [38,44]. In this study, we investigated the in vivo effect of toluene using female Wistar rats. We found that exposure to a low dose (2000 ppm) significantly increased body weight, whereas no effect was observed with the middle and high doses. The effect of toluene exposure on animal body weight varies in the literature. While some studies reported a decrease in body weight, others reported an increase in body weight following exposure to toluene [45]. This variation may be due to many factors, including the method of administration, whether it was administered through gavage, injection or inhalation [46]. 

In the present study, we identified the reproductive toxic effect of toluene exposure on ovarian function and follicle development potential and initially confirmed that this toxicity was derived from ovarian structure disruption, inhibition of folliculogenesis and steroidogenesis-related markers, induction of apoptosis and autophagy. We first observed a reduced ovarian weight in rats treated with doses of 4000 and 8000 ppm. This reduction was associated with an increase in the number of abnormal follicles, whereas the number of growing follicles was reduced at 8000 ppm. Our results are consistent with previous studies that reported that toluene exposure caused multiple injuries in adult female mouse ovaries, leading to the disruption of the follicular development process and altering their histological structure [47,48]. Based on the fact that hydrocarbons are considered endocrine disruptors, we analyzed some reproductive hormones that have a direct effect on ovarian function. We found that toluene exposure caused a significant increase in the secretion of the hormones progesterone and testosterone when compared to the control group. Based on the fact that the steroidogenic factor *Cyp17a* is responsible for androgen production, including testosterone [49,50], this increase in testosterone levels is in accordance with the significant increase in *Cyp17a* mRNA levels shown by RT–PCR. The increase in progesterone release discords the results of our previous study in which we used cultured ovarian granulosa cells, in which we found that the addition of toluene resulted in suppression of progesterone secretion [39]. It has been shown that progesterone may have antioxidative and protective effects since it inhibits the production of oxygen species (ROS) in the ovaries of benzene-treated rats [51] and protects against bropirimine-mediated embryolethality [52]. Thus, the upward trend of progesterone with increasing doses of toluene may be interpreted as a strategy of protection created by the ovary against toluene exposure and not due to the increase in the number of the corpus luteum which has the secretion of progesterone as its primary function. This significant increase in progesterone could also be due to the effect of other factors, since the corpora lutea are also under the control of other organs rather than the ovary, including the hypothalamus and pituitary and even the uterus [53]. Indeed, a previous study showed that toluene caused a decrease in the level of follicle stimulating hormone (FSH) responsible for the growth of ovarian follicles [54], which may have, as a consequence, an increase in progesterone as a result of the feedback interactions between ovarian and gonadotropic hormones. Intestinally, it has been previously reported that progesterone activates autophagy as a strategic neuroprotective mechanism [55] which may suggest that the significant progesterone release after toluene exposure has a protective effect of ovarian cells through triggering autophagy. 

During follicular growth and development, *Insl3*, *Ccnd2*, *Actb* and *Gdf* are essential for granulosa cell proliferation and differentiation [56]. Our data show that toluene exposure caused a significant downward trend in the mRNA levels of these genes with increasing doses, which may have negatively affected granulosa cell proliferation and follicle development. In particular, INSL3 in females of Mammalia is produced from ovarian follicular theca cells [57] and plays a role in the control of the number of healthy growing follicles [58,59]. The downregulation of this gene under the effect of toluene exposure may have increased the number of abnormal follicles. Among the positive regulators of granulosa cell proliferation are D-type cyclins (CCND1, CCND2 and CCND3) that activate the cyclin-dependent kinases CDK 4 and 6 [60]. The knockout of cyclin D2 (Ccnd2) led to the impairment of granulosa cell proliferation and the failure of ovulation [61], which is in accordance with our results. Interestingly, toluene exposure caused a significant decrease in the expression of the GDF protein, which negatively affected the number of developing follicles, mainly at 8000 ppm. This is evident since GDF-9 is needed for early stages of follicle development in several mammalian species [62] by suppressing granulosa cell apoptosis and follicular atresia [63]. In other words, the downregulation of GDF-9 increases the occurrence of apoptosis in ovarian cells. This is in accordance with the TUNEL assay results, which demonstrated that apoptotic cells increased in a dose-dependent manner in response to toluene exposure, even though the increase was not significant at 2000 ppm. Consistent with previous studies, environmental toxins, including benzene, toluene and xylene, have been shown to cause cellular processes to be disrupted by inducing apoptosis and genotoxicity [10,64,65,66,67,68,69]. 

Autophagy is an evolutionarily conserved cellular mechanism that contributes to the protection of cells against a variety of intracellular and extracellular stressors [70]. It is an important process that may support cell survival or trigger cell death pathways [71,72]. Our immunofluorescence results showed that exposure to toluene significantly increased the protein expression of LC3 and its encoding gene for all doses of treatment. On the other hand, toluene exposure triggered apoptosis for middle and high doses of treatment (4000 and 8000 ppm) whereas TMR-Red increase was not significant in ovaries from females treated with the low dose (2000 ppm). These results suggest that a low dose of toluene exposure may trigger the autophagy machinery which worked to protect ovarian cells from the damage by inhibiting apoptosis. However, the activated autophagy after treatment with both middle and high doses was associated with facilitation of apoptosis rather than its repression. Our results are consistent with what was mentioned about the role of environmental pollutants, especially industrial compounds, that are able to trigger autophagy, either by increasing it as a protective response against cellular damage or by a pathway to shift its protective role toward a pro-cell death mechanism [70]. In fact, it has been previously shown that autophagy and apoptosis are two closely interconnected processes that can occur simultaneously and share many factors in response to diverse stimuli [72,73]. In some cases of low-dose toxins, the activation of autophagy appears to be an initial event in the cell self-repair mechanism that protects cells against damage, leading to the inhibition of apoptosis [74]. Once unable to protect cells against stressful conditions, autophagy upregulates the apoptotic process, leading to cell death [75], which is in accordance with our TUNEL assay data. 

## 5. Conclusions

We conclude from our in vivo study that toluene disrupted ovarian function injury by significantly decreasing the number of growing follicles and increasing the number of abnormal follicles, progesterone and testosterone levels. In addition, it inhibited GDF-9 protein expression and mRNA levels of genes responsible of follicular growth and development. At lower concentrations of toluene exposure, autophagy was activated to prevent ovarian injury by inhibiting apoptosis. Middle and higher concentrations of toluene induced autophagy and apoptosis that eventually caused ovarian cell death. Our findings provide novel information on the mechanism of disruption of hydrocarbons, and the potential mutual interactions between autophagy and apoptosis in reproductive toxicity that will aid in future research. For future studies, it will be critical to determine the in utero impacts of toluene exposure in order to determine the alterations and potential consequences that could have a long-term impact on the health of future generations.

## Figures and Tables

**Table 1:** Primers for real-time RT-PCR.

Gene Symbol		Sequences
***INSL3***	Forward:	CTTCCTCACCAGGCTTCTCA
	Reverse:	CACCACCTGAGCCCTACAAT
***CYP17a1***	Forward:	ACTGAGGGTATCGTGGATGC
	Reverse:	TCGAACTTCTCCCTGCACTT
***CYP1*** ***9***	Forward:	TGAGTCTCCCAAGGTCATCC
	Reverse:	GGGTTCAGCATTTCCAAAAA
***LHR***	Forward:	TTATTCCGCCATCTTTGAGG
	Reverse:	ACAGGGGTTGAAAGCATCTG
***CCND2***	Forward:	CCTCACGACTTCATTGAGCA
	Reverse:	GGTAGCACACAGAGCGATGA
***IGF-I***	Forward:	CCGCTGAAGCCTACAAAGTC
	Reverse:	GGGAGGCTCCTCCTACATTC
***ESR1***	Forward:	CATCGATAAGAACCGGAGGA
	Reverse:	AAGGTTGGCAGCTCTCATGT
***ESR2***	Forward:	GAAGCTGAACCACCCAATGT
	Reverse:	CAGTCCCACCATTAGCACCT
***A*** ***CTB***	Forward:	AGCCATGTACGTAGCCATCC
	Reverse:	ACCCTCATAGATGGGCACAG
***GDF9***	Forward:	GATGTGACCTCCCTCCTTCA
	Reverse:	GCCTGGGTACTCGTGTCATT
***ATG5***	Forward:	CCTGAAGACGGAGAGAAGAAGAG
	Reverse:	CGGGAAGCAAGGGTGTCAT
***LC3***	Forward:	TGTTAGGCTTGCTCTTTTGG
	Reverse:	GCAGAGGAAATGACCACAGAT

## Data Availability

The data that support the findings of this study are available from the corresponding author, (Abdel Halim Harrath), upon reasonable request.

## Abbreviations

Actb	Actin Beta
Atg5	Autophagy related 5
BSA	Bovine serum albumin
cDNA	Complementary DNA
Ccnd1	Cyclin D1
Ccnd2	Cyclin D2
Cdk	Cyclin-dependent kinases
Cyp17a	Cytochrome P450 family 17 subfamily A
Cyp19	Cytochrome P450 family 19
Esr1	Estrogen receptor 1
Esr2	Estrogen receptor 2
FITC	fluorescein isothiocyanate
Gdf-9	Growth differentiation factor 9
Igf-1	Insulin-like growth factor-1
Insl3	Insulin-Like Peptide 3
Lhr	Luteinizing hormone receptor
LC3	Microtubule-associated protein 1A/1B-light chain 3
NBF	Neutral buffered formalin
PBS	Phosphate-buffered saline
RT-PCR	Real-time PCR
TUNEL	Terminal deoxynucleotidyl transferase mediated nick end-labeling
TdT	Terminal deoxynucleotidyl transferase
